# Supplementary material for: Chronic Voluntary Alcohol Consumption Alters Promoter Methylation and Expression of Fgf-2 and Fgfr1
Source: Int J Mol Sci. 2023 Feb 7;24(4):3336. doi: 10.3390/ijms24043336 (PMC9963845; doi:10.3390/ijms24043336)
Supplement: Supplementary file 1 [file ijms-24-03336-s001.zip › Revision_Herburg et al., 2023_Supplementary files.pdf]

Supplementary Materials:

Supplemental Table S1. Analyzed C57BL/6J animals for this study. Body weights were determined at the beginning of the experiments.

Animal number	Start body weight (g)	Group
421	24.98	Alcohol
422	26.6	Alcohol
423	24.14	Alcohol
442	28.67	Control
443	28.93	Control
444	28.29	Alcohol
445	28.96	Alcohol
477	27.75	Control
478	26.91	Control
479	25.48	Control
480	27.89	Alcohol
481	28.81	Alcohol
482	26.54	Control
483	29.53	Control
484	26.13	Control
485	26.53	Alcohol
486	25.31	Alcohol
487	28.37	Alcohol

Supplemental Table S2. *Fgf-2* and *Fgfr1* primer sequences for methylation analysis. *Fgf-2* (fibroblast growth factor 2), *Fgfr1* (fibroblast growth factor receptor 1), TSS (transcription start site, first base of exon 1), Tm (melting temperature).

Primer name	Primer sequence (5′ to 3′)	Position counting from TSS (bp)	Tm
Fgf-2 F3	TAAGAAATATTTAATAATTGAGG	-121	50.7 °C
Fgf-2 R2	TAACAACTCTTAAAACTCCC	+475	
Fgf-2 F4	GTTGTATTGGGTTGGGAGTT	+360	58 °C
Fgf-2 R4	TCTTAATATCCCAAATCCCC	+757	
Fgfr1 F1	GAAAAGAATTATTGGAGTTAGGTGT	-629	58 °C
Fgfr1 R1	CTAATTTTTCCACCTCCCTTC	+28	

Supplemental Table S3. Sequences of primers used for qRT-PCR (quantitative reverse transcription polymerase chain reaction).

Gene	Primer sequence	Product size	Melting point
Fgf-2	5′-GAGAAGAGCGACCCACACG-3′<br>5′-GGCACACACTCCCTTGATAGA-3′	78 bp	77 °C
Fgfr1	5′-CTTGCCGTATGTCCAGATCC-3′<br>5′-TCCGTAGATGAAGCACCTCC-3′	77 bp	76 °C
Gapdh	5′-GAACATCATCCCTGCATCCA-3′<br>5′-GAACATCATCCCTGCATCCA-3′	78 bp	81.1 °C

Supplemental Table S1. Statistics of significant CpG positions of the *Fgf-2* gene. Two-tailed *t*-tests were performed with Prism 8.0; t (test statistic), df (degrees of freedom); PFC (prefrontal cortex), NAc (nucleus accumbens), DLS (dorsolateral striatum), DMS (dorsomedial striatum), VTA (ventral tegmental area).

		***t*-Test for Equality of Means**		
**Tissue**	**CpG position**	**t**	**df**	***p*-value**
Blood	+174	-2.397	11	0.03
PFC	+555	2.114	18	0.05
	+660	2.447	18	0.02
	+700	2.463	13.2	0.03
NAC	+342	-2.237	16	0.04
	+381	-2.097	18	0.05
	+552	2.438	18	0.03
DLS	+268	-2.450	17	0.03
	+552	-2.910	18	0.01
	+702	2.347	18	0.03
DMS	+278	-2.590	16	0.02
	+307	-2.213	15	0.04
VTA	+558	2.681	18	0.02
	+609	2.913	18	0.01
	+636	2.305	18	0.03
	+639	2.965	18	0.01
	+643	2.333	18	0.03
	+669	2.435	18	0.03

Supplemental Table S2. Fixed effects of factors and covariates of the MLM model with *Fgf-2* methylation as dependent variable. Df (degrees of freedom), ddCT (delta-delta Ct).

Source	Numerator df	Denominator df	F-statistics	Significance
Intercept	1	6064	2591.09	0.00
CpG position	65	6064	67.69	0.00
Tissue	5	6064	56.89	$5.2x10^{-58}$
Group	1	6064	76.36	$3x10^{-18}$
FGF2_ddCT	1	6064	9.84	0.002
Tissue * Group	5	6064	25.11	$3.9x10^{-25}$
Tissue*Group * FGF2_ddCT	11	6064	22.56	$6.8x10^{-46}$

Supplemental Table S6. Statistics of significant CpG positions of the *Fgfr1* gene. Two-tailed *t*-tests were performed with Prism 8.0; t (test statistic), df (degrees of freedom); PFC (prefrontal cortex), DLS (dorsolateral striatum).

		***t*-Test for Equality of Means**		
**Tissue**	**Position**	**t**	**df**	***p*-value**
PFC	-270	3.740	18	0.002
DLS	-361	2.210	17	0.04

Supplemental Table S7. Prediction of transcription factor binding sites within the *Fgfr1* promoter; PFC prefrontal cortex), DLS (dorsolateral striatum). Factorbook was used for binding site prediction (www.factorbook.org).

Transcription factor	Brain region (CpG position)	Consensus sequence
Nuclear Factor Erythroid 2 (NFE2)	PFC (-270 bp)	5′-ATGA[C/G]TCA-3
Specificity Protein 1 (SP1)	DLS (-361 bp)	5′-CCCCGCCCCC-3′
Zinc Finger Protein 460 (ZNF460)	DLS (-361 bp)	5′-GCCTCCCG-3

Supplemental Table S8. Fixed effects of factors and covariates of the MLM model with *Fgfr1* methylation as dependent variable. Df (degrees of freedom), ddCT (delta-delta Ct).

Source	Numerator df	Denominator df	F-statistics	Significance
Intercept	1	687	52830.23	0.00
CpG position	6	687	827.03	0.00
Tissue	5	687	9.43	$1x10^{-8}$
Group	1	687	0.21	0.646
FGFR1_ddCT	1	687	0.76	0.384
Tissue * Group	5	687	1.57	0.166
Tissue * Group * FGFR1_ddCT	11	687	1.28	0.229

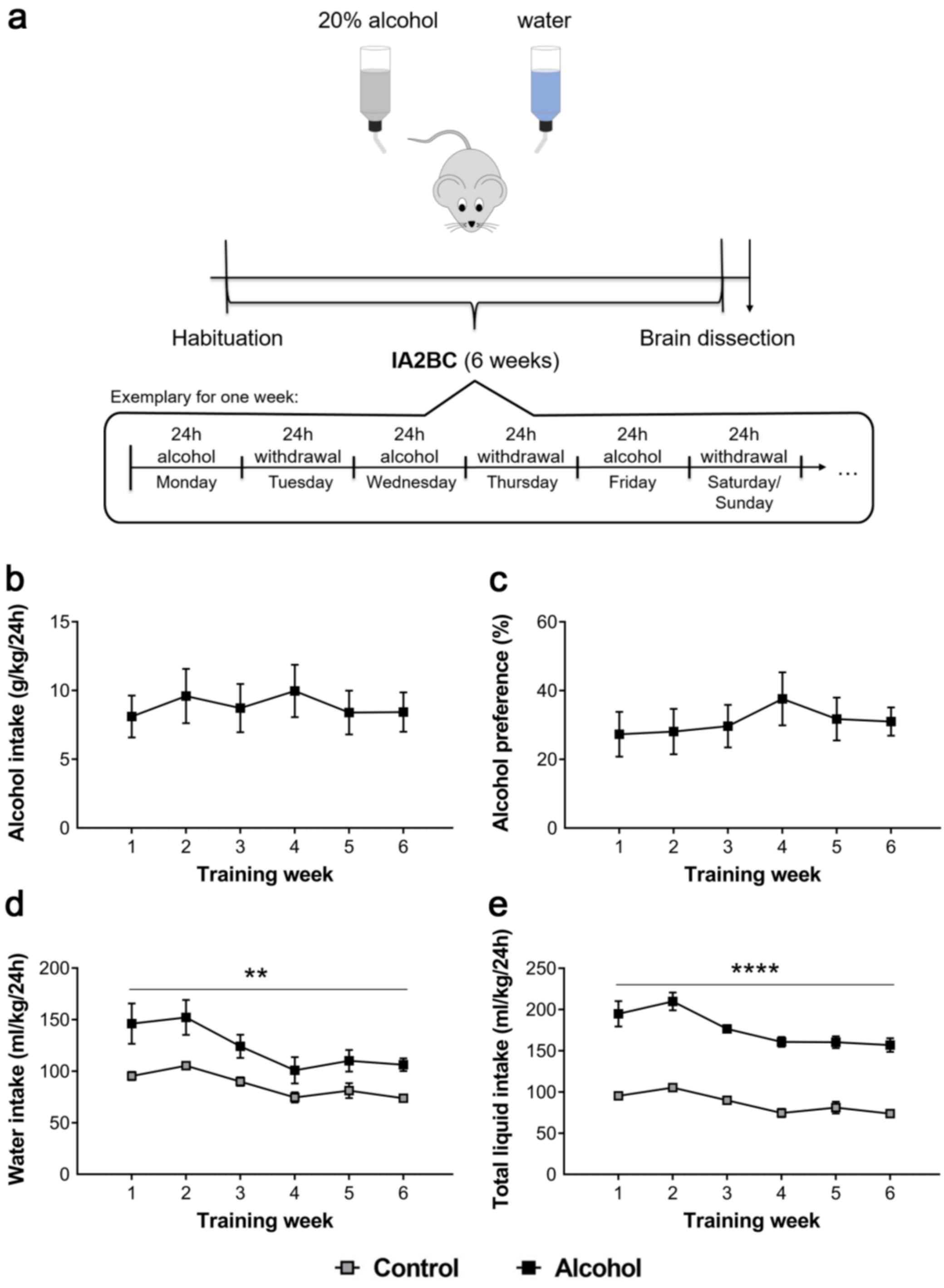

Supplemental Figure S1. Experimental timeline scheme of the IA2BC (intermittent access to 20 % alcohol in a two-bottle choice) procedure. Mice were trained to consume alcohol in the IA2BC procedure during a six-week period (a). Alcohol consumption (g/kg/24h; b) and preference (c), water intake (ml/kg/24h; d), and total liquid intake (ml/kg/24h; e) were measured. Data are presented as mean ± SEM (standard error of the mean), two-way mixed-model ANOVA; n=10 **$p \leq 0.01$; ****$p \leq 0.0001$ compared to control group.

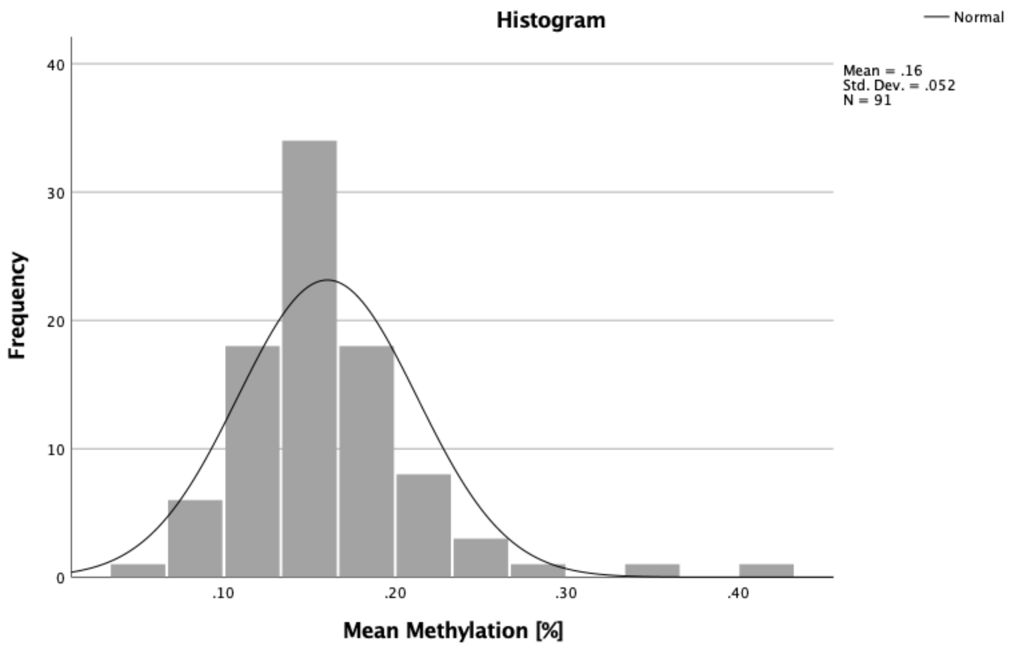

Supplemental Figure S2. Distribution of methylation measurements for normality assessment.

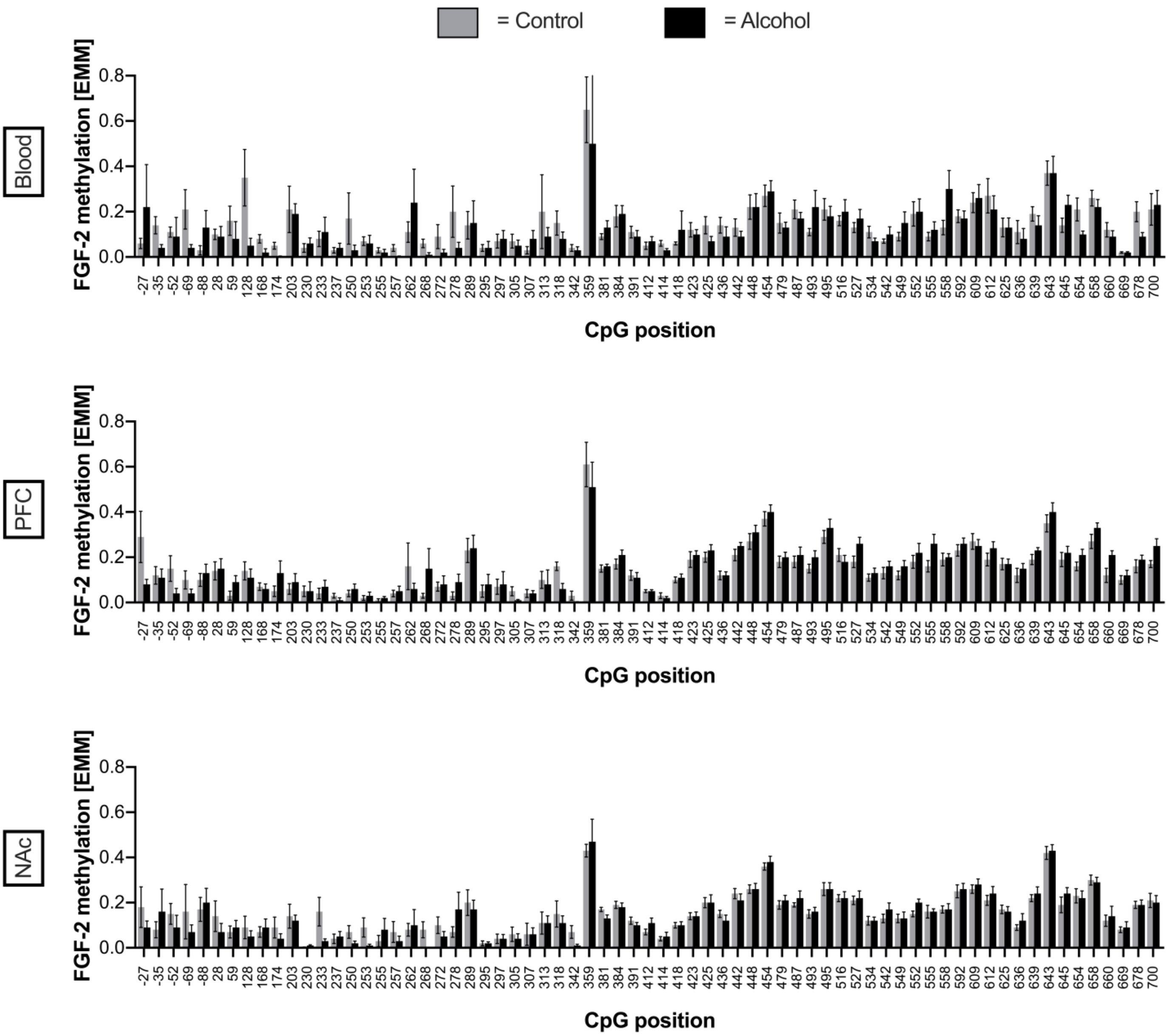

Supplemental Figure S3. Alterations in the methylation of *Fgf-2* after alcohol consumption in the blood, PFC (prefrontal cortex), and NAc (Nucleus accumbens). Estimated marginal means of the methylation rate of all 70 examined CpG-sites of the *Fgf-2* promoter in tissue samples after chronic alcohol exposure. Data are represented as mean ± SEM (standard error of the mean) (n=10 biological replicates).

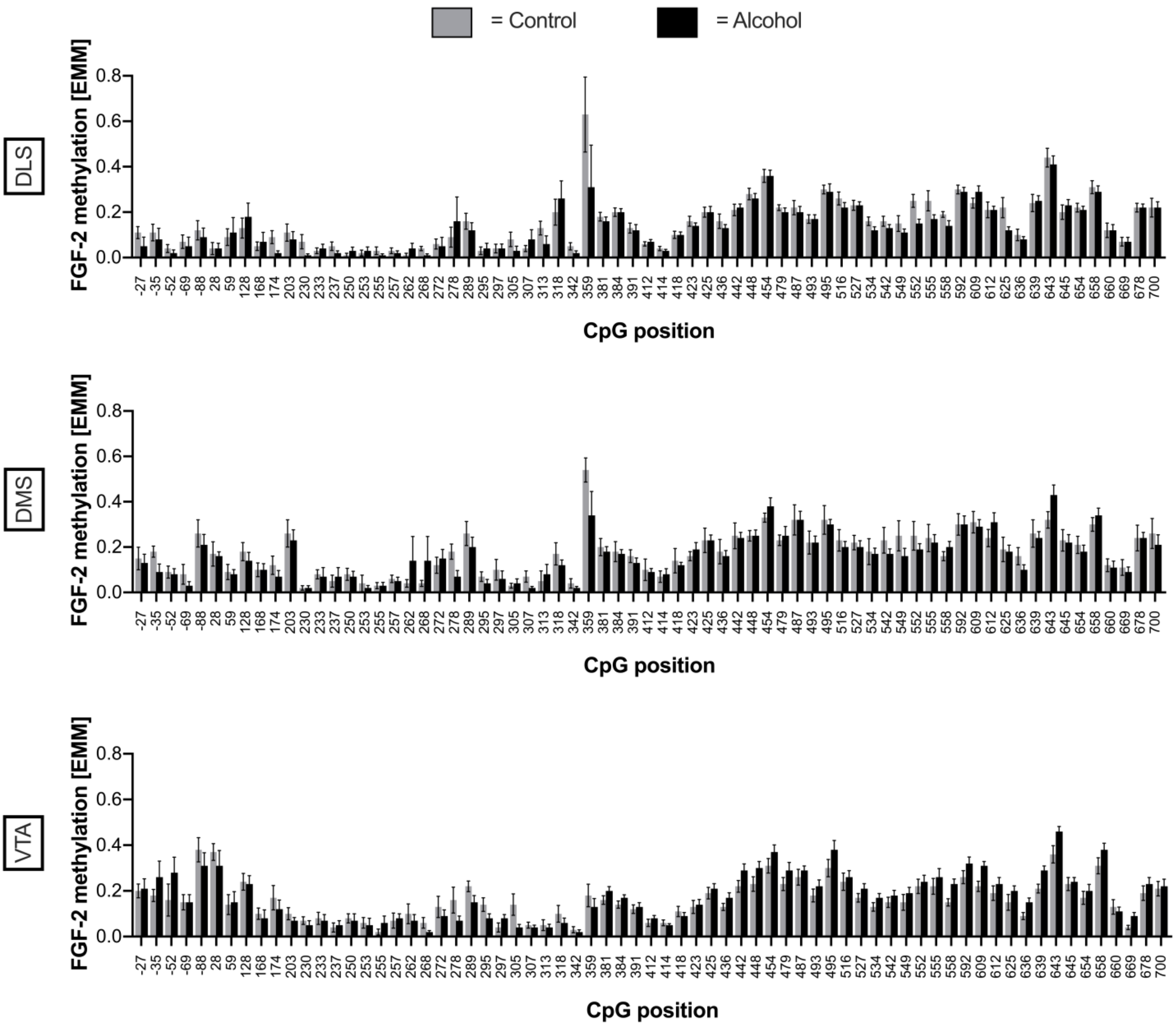

Supplemental Figure S4. Alterations in the methylation of *Fgf-2* after alcohol consumption in DLS (dorsolateral striatum), DMS (dorsomedial striatum), and VTA (ventral tegmental area). Estimated marginal means of the methylation rate of all 70 examined CpG-sites of the *Fgf-2* promoter in tissue samples after chronic alcohol exposure. Data are represented as mean ± SEM (n=10 biological replicates).

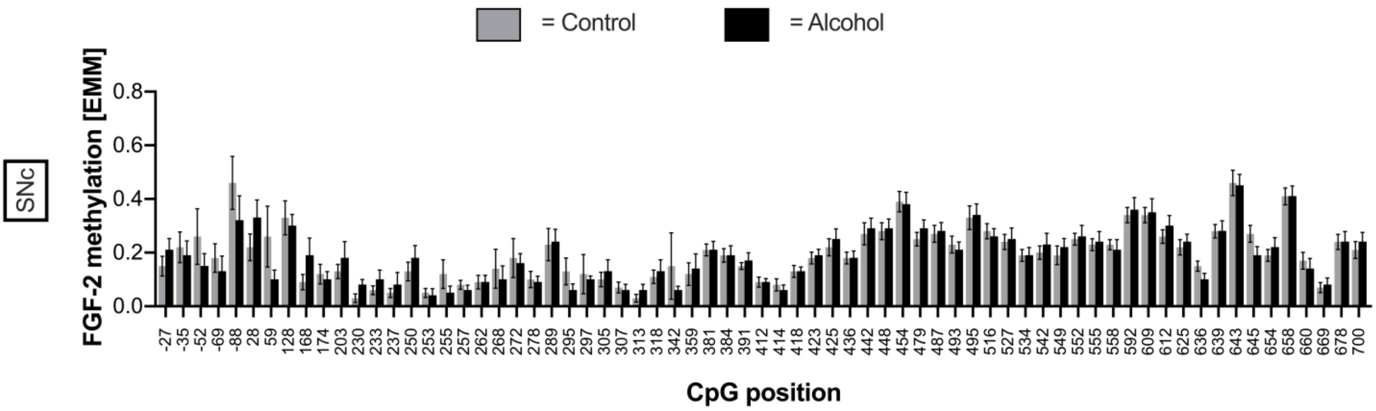

Supplemental Figure S5. Alterations in the methylation of *Fgf-2* after alcohol consumption in the SNc (substantia nigra). Estimated marginal means of the methylation rate of all 70 examined CpG-sites of the *Fgf-2* promoter in tissue samples after chronic alcohol exposure. Data are represented as mean ± SEM (n=10 biological replicates).

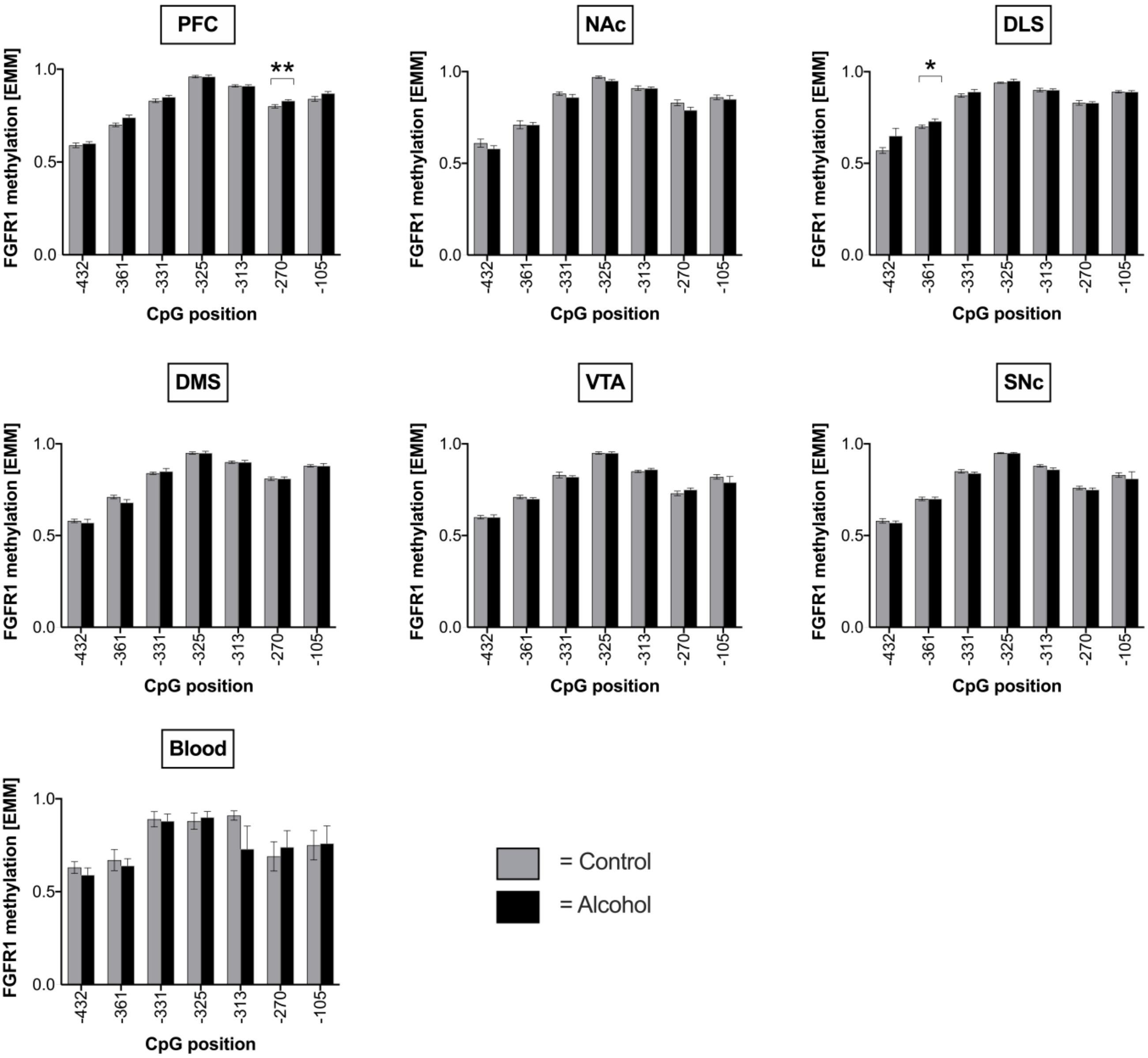

Supplemental Figure S6. Alterations in the methylation of *Fgfr1* after alcohol consumption. Estimated marginal means of the methylation rate of all seven examined CpG-sites of the *Fgfr1* promoter in the PFC, NAc, DLS, DMS, VTA, SNc, and blood tissue samples after alcohol (20 % alcohol during a six-week period) exposure. Data are represented as mean ± SEM (n=10 biological replicates) with a two-tailed Student's t-test, **p*≤0.05; ** *p*≤0.01 compared to control group.
