# Supplementary material for: Chronic Voluntary Alcohol Consumption Alters Promoter Methylation and Expression of Fgf-2 and Fgfr1
Source: Int J Mol Sci. 2023 Feb 7;24(4):3336. doi: 10.3390/ijms24043336 (PMC9963845; doi:10.3390/ijms24043336)
Supplement: Supplementary file 1 [file ijms-24-03336-s001.zip › Supplemental Excel File S1.pdf]

Transcription factor	Tissue (CpG position)	Binding motif	Publications (keywords entered in GeneCards)			
			FGF2, FGFR1, growth factors	Alcohol, abuse, addiction, drugs	Methylation, epigenetic	PFC, Nac, DLS, DMS, VTA, SNc, striatal, mesolimbic, reward
**Arnt**<br>Aryl hydrocarbon receptor nuclear translocator	DLS (702)	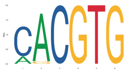	Mizuta et al., 2008	Attignon et al., 2017<br>Morris et al., 2018		
**Arnt2**<br>Aryl Hydrocarbon Receptor Nuclear Translocator 2	DLS (702)	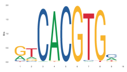			Yin et al., 2017	
**ASCL2**<br>Achaete-Scute Family BHLH Transcription Factor 2	VTA (643)	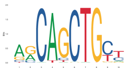	Hughes et al., 2004		Yin et al., 2017	
**ATF1**<br>Activating Transcription Factor 1	VTA (639)	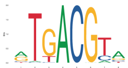	Tan et al., 1996			
**ATF3**<br>Activating Transcription Factor 3	DMS (307)<br>VTA (639)	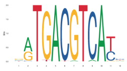	Tan et al., 1994	Mallory et al., 2005; Jutooru et al., 2009		
**CREB1**<br>CAMP Responsive Element Binding Protein	VTA (639)	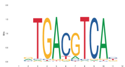	Tan et al., 1996; Stachowiak et al., 2003	Tabakoff et al., 2009;<br>Schumann et al., 2008;<br>Chen et al., 2015;<br>Chanda et al., 2013;<br>Paul et al., 2010;<br>Mahajan et al., 2009; Pandey et al., 2004;<br>Blendy et al., 1998; Wolf et al., 2016; Dinieri et al., 2009; Nestler er al., 2006; Jaworski et al., 2006; Olson et al., 2005		Dinieri et al., 2009; Nestler er al., 2006; Jaworski et al., 2006; Olson et al., 2005
**CREB3L1**<br>Cyclic AMP-responsive element-binding protein 3-like protein 1	DLS (307)	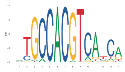	Zhu et al., 2016			
**CREM**<br>CAMP Responsive Element Modulator	VTA (639)	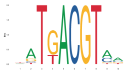		Miller et al., 2018	Yin et al., 2017	Miller et al., 2018
**CTCFL**<br>CCCTC-Binding Factor Like	DLS (268; 307)<br>VTA (669)	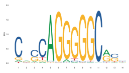				
**Ddit3**<br>DNA damage-inducible transcript 3 protein<br>**Cebpa**<br>CCAAT/enhancer-binding protein alpha	Blood (174bp)	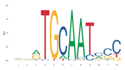				
**E2F1**<br>E2F Transcription Factor 1	DLS (268)<br>NAc (342)<br>VTA (636)<br>PFC (700)	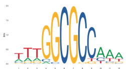	Zhou et al., 2020; Kanai et al., 2009; Parakati et al., 2005; Stapelberg et al., 2004	Major et al., 2017	Yin et al., 2017	
**E2F6**<br>E2F Transcription Factor 6	VTA (636)	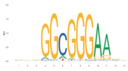				
**EGR1**<br>Early Growth Response 1	DLS (268;702)<br>VTA (588)<br>PFC (660, 700)	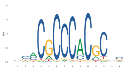	Kundumani-Sridharan et al., 2010; Fahmy et al., 2003; Shi et al., 2018; Passiatore et al., 2011; Nentwich et al., 2009; Perez-Castro et al., 1997; Bhindi et al., 2005; Midgley et al., 2004; Jin et al., 2000;	Li et al., 2019; Pritchard et al., 2005; Shin et al., 2021; Thomes et al., 2013; Quiñones et al., 2003;	Zandarashvili et al., 2015;	
**ELF1**<br>E74 Like ETS Transcription Factor 1	PFC (660)	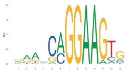			Yin et al., 2017	
**ELF2**<br>E74 Like ETS Transcription Factor 2	Nac (342)	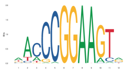			Yin et al., 2017	
**ELK4**<br>ETS Transcription Factor ELK4	NAc (342)	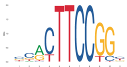			Yin et al., 2017	

Transcription factor	Tissue (CpG position)	Binding motif	Publications (keywords entered in GeneCards)			
			FGF2, FGFR1, growth factors	Alcohol, abuse, addiction, drugs	Methylation, epigenetic	PFC, Nac, DLS, DMS, VTA, SNc, striatal, mesolimbic, reward
**Fli1**<br>Friend Leukemia Integration 1 Transcription Factor	NAc (342)	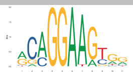			Yin et al., 2017	
**FOS**<br>Fos Proto-Oncogene, AP-1 Transcription Factor Subunit	DLS (268)	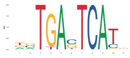	Kim et al., 2003; Hofer et al., 2005; Weekes et al., 2016; Lee et al., 2013; Rosenthal et al., 2005; Wang et al., 2004; Li et al., 2010; Kang et al., 2005; Taniguchi et al., 2003	Samaha et al., 2004; Tabakoff et al., 2009; Bichenkov et al., 2009; Raatesalmi et al., 2002; Alling et al., 1994; Alling et al., 1993; Valjent et al., 2000		Johnson et al., 2010; Perrotti et al., 2004; Valjent et al., 2000
**Gfi1b**<br>Growth Factor Independent 1B Transcription Repressor	Blood (174bp)	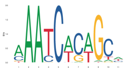				
**HINFP**<br>Histone H4 Transcription Factor	DMS (307)<br>PFC(660)	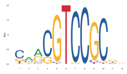			Pulukuri et al., 2006	
**KLF1**<br>Kruppel Like Factor 1	PFC (700)	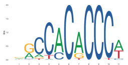				
**KLF12**<br>Kruppel Like Factor 12	DMS (278)	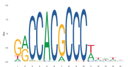			Yin et al., 2017	
**KLF15**<br>Kruppel Like Factor 15	DMS (278)	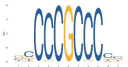			Yin et al., 2017	
**MAX**<br>MYC Associated Factor X	DLS (702)	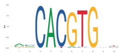				
**MAZ**<br>Myc-associated zinc finger protein	VTA (558)	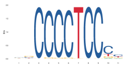				
**MBD2**<br>Methyl-CpG Binding Domain Protein 2	DMS (278)<br>NAc (381)			Bönsch et al., 2006	Ghoshal et al., 2004; Jin et al., 2005; Tan et al., 2006; Xing et al., 2008	
**MXI1**<br>MAX Interactor 1, Dimerization Protein	DLS (702)	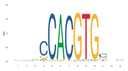				
**MYC**<br>MYC Proto-Oncogene, BHLH Transcription Factor	VTA (643)<br>DLS (702)	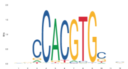	Park et al., 2020; Ronca et al., 2020; Azarnezhad et al., 2018; Liu et al., 2017; Park et al., 2016; Xue et al., 2015; Bhattacharyya et al., 2020;	Nakahara et al., 2003; Sauders et al., 1995a; Sauders et al., 1995b		
**MYCN**<br>N-myc proto-oncogene protein	DMS (307)<br>DLS (702)<br>PFC(660)	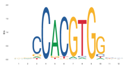				
**NRF1**<br>Nuclear Respiratory Factor 1	DLS (268)<br>VTA (588)<br>PFC (660)					
**RUNX3**<br>Runt-Related Transcription Factor 3	Blood (174)	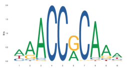				
**SP1**<br>Specificity Protein 1	DMS (278)		Shi et al., 2016M Chan et al., 2010; Wang et al., 2010; Waters et al., 2009; Boer et al., 2005; Perez-Castro et al., 1997; Bonello et al., 2004; Chen et al., 2017; Chang et al., 2014; Benjamin et al., 2010; Midgley et al., 2004; Cole et al., 1997; Bermudez et al., 2008	Hedrick et al., 2016; Norkina et al., 2007; Hur et al., 1992; Edenberg et al., 1992; Do et al., 2013; Zhang et al., 2015;	Furuta et al., 2008; Mudduluru et al., 2008; Liu et al., 2008; Yu et al., 2020; Norkina et al., 2007	

Transcription factor	Tissue (CpG position)	Binding motif	Publications (keywords entered in GeneCards) FGF2, FGFR1, growth factors	Alcohol, abuse, addiction, drugs	Methylation, epigenetic	PFC, Nac, DLS, DMS, VTA, SNc, striatal, mesolimbic, reward
**SP3**<br>Specificity Protein 3	PFC (700)			Gromnicova et al., 2012		
**SPDEF**<br>SAM Pointed Domain Containing ETS Transcription Factor	NAc (381)	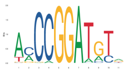		Tay et al., 2019	Yin et al., 2017	
**TAF1**<br>TATA-Box Binding Protein Associated Factor 1	DMS (307)<br>VTA (639; 643)<br>PFC (660)					
**TYY1**<br>Transcriptional Repressor Protein YY1	Nac (552)<br>DLS (552)<br>VTA (558; 609)	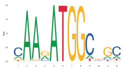		Hur et al., 1992	Yin et al., 2017; Buira et al., 2010; Zhang et al., 2016; Mahadevan et al., 2014	
**YY2**<br>Transcription Factor Yin Yang 2	NAc (552)<br>DLS (552)<br>VTA (558)	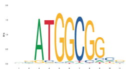			Yin et al., 2017	
**ZBED1**<br>Zinc Finger BED-Type Containing 1	VTA (636)	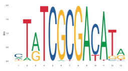			Yin et al., 2017	
**ZBTB12**<br>Zinc Finger And BTB Domain Containing 12	VTA (609)	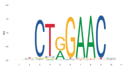				
**ZBTB14**<br>Zinc Finger And BTB Domain Containing 14	DLS (268)<br>NAc (342; 381)<br>PFC (660)<br>VTA (669)	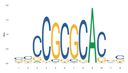			Yin et al., 2017	
**ZBTB33**<br>Zinc Finger And BTB Domain Containing 33	VTA (636)<br>PFC (660)	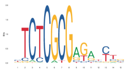				
**Zic2**<br>Zinc finger protein 2	Blood (174)	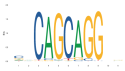				
**ZNF317**<br>Zinc finger protein 317	Blood (174)	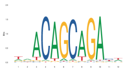				
**ZNF341**<br>Zinc Finger Protein 341	VTA (669)	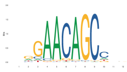				
**ZNF460**<br>Zinc Finger Protein 460	VTA (558)	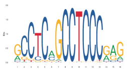			Yin et al., 2017	
