# Supplementary material for: Chronic Voluntary Alcohol Consumption Alters Promoter Methylation and Expression of Fgf-2 and Fgfr1
Source: Int J Mol Sci. 2023 Feb 7;24(4):3336. doi: 10.3390/ijms24043336 (PMC9963845; doi:10.3390/ijms24043336)
Supplement: Supplementary file 1 [file ijms-24-03336-s001.zip › Supplemental Excel File S2.pdf]

Transcription factor	Tissue (CpG position)	Binding motif	Publications (keywords entered in GeneCards)			
			FGF2, FGFR1, growth factors	Alcohol, abuse, addiction, drugs	Methylation, epigenetic	PFC, Nac, DLS, DMS, VTA, SNc, striatal, mesolimbic, reward
**NFE2**<br>Nuclear Factor, Erythroid 2	PFC (-270bp)	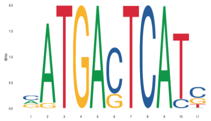			Yin et al., 2017;<br>Kiekhaefer et al., 2002	
**SP1**<br>Transcription Factor/Specificity Protein 1	DLS (-361)	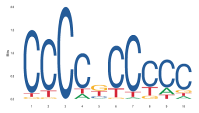	Shi et al., 2016M Chan et al., 2010; Wang et al., 2010; Waters et al., 2009; Boer et al., 2005; Perez-Castro et al., 1997; Bonello et al., 2004; Chen et al., 2017; Chang et al., 2014; Benjamin et al., 2010; Midgley et al., 2004; Cole et al., 1997; Bermudez et al., 2008	Hedrick et al., 2016; Norkina et al., 2007; Hur et al., 1992; Edenberg et al., 1992; Do et al., 2013; Zhang et al., 2015;	Furuta et al., 2008; Mudduluru et al., 2008; Liu et al., 2008; Yu et al., 2020; Norkina et al., 2007	
**SP2**<br>Transcription Factor/Specificity Protein 2	DLS (-361)	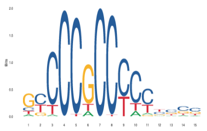			Yin et al., 2017	
**SP3**<br>Transcription Factor/Specificity Protein 3	DLS (-361)	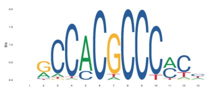		Gromnicova et al., 2012		
**ZNF460**<br>Zinc Finger Protein 460	DLS (-361)	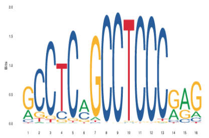			Yin et al., 2017	
**ZNF770**<br>Zinc Finger Protein 770	DLS (-361)	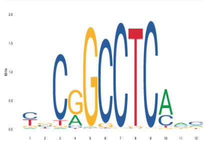				
